# Can Cuticular Hydrocarbons Be used as Chemotaxonomic Tool for *Neosilba* McAlpine (Diptera: Lonchaeidae)?

**DOI:** 10.1007/s10886-026-01693-8

**Published:** 2026-02-24

**Authors:** Jean Carlos dos Santos Lima, Laura Jane Gisloti, Sidnei Eduardo Lima-Junior

**Affiliations:** 1 Programa de Pós-Graduação em Entomologia e Conservação da Biodiversidade, Universidade Federal da Grande Dourados, Dourados, MS Brazil; 2 Laboratório de Entomologia, Instituto de Biodiversidade e Florestas, Universidade Federal do Oeste do Pará, Santarém, PA Brazil; 3 Centro de Estudos em Recursos Naturais, Universidade Estadual de Mato Grosso do Sul, Dourados, MS Brazil

**Keywords:** Chemotaxonomy, Fruit flies, Lance flies, Surface lipids, Tephritoidea

## Abstract

Recent studies have shown an interest in the *Neosilba* McAlpine (Diptera: Lonchaeidae) genus, as its larvae are reported to feed on many commercially relevant fruits. However, the taxonomical difficulty to identify the species of this genus apart ends up hampering and discouraging research on these flies. In this work, cuticular hydrocarbons were extracted, identified, and evaluated as chemotaxonomic characters from six species of adult *Neosilba* lance flies. We also investigated whether the fruits used by these flies as larval nutrition substrates influence the cuticular hydrocarbon profiles expressed by these flies. Forty-one hydrocarbon components were identified by gas chromatography-mass spectrometry. The components represented four classes: *n*-alkanes, monomethyl alkanes, dimethyl alkanes and alkenes. Cuticular hydrocarbon analysis does not provide a reliable method to differentiate *Neosilba* species. The CHC components were overall common but the blend ratios differed even within *Neosilba* species. Cuticular hydrocarbon mixtures show host fruit-associated variation. The expression of cuticular hydrocarbons dependent on host fruit found in this study may suggest the formation of host race.

**Supplementary Information:**

The online version contains supplementary material available at 10.1007/s10886-026-01693-8.

## Introduction

Cuticular hydrocarbons (CHCs) are compounds that cover the cuticle surface of all insects (Blomquist and Bagneres 2010). Although their initial function was to serve as a protective barrier against water loss to the environment and the entry of microorganisms through the cuticle, they have been repurposed through evolution for intraspecific recognition (Blomquist and Bagnères 2010; Menzel et al. 2019; Sprenger and Menzel 2020).

The cuticle represents a complex mixture of different types of CHCs, such as linear alkanes, branched alkanes, and unsaturated hydrocarbons (Drijfhout et al. 2009; Blomquist and Ginzel 2021). Each type of CHC differs in structure and consequently in its function on the cuticle (Drijfhout et al. 2009). For example, linear alkanes are highly aggregated molecules due to Van der Waals forces, resulting in a high melting point, making them excellent waterproofing agents (Sprenger et al. 2021). This is corroborated by studies exposing insects to high temperatures and/or low humidity, which induce an increase in the amount of this class on the cuticle (Wagner et al. 2001; Michelutti et al. 2018; Duarte et al. 2019).

The presence of methyl groups or double bonds imparts fluidity to CHC molecules, and their low melting points make them less effective in waterproofing the cuticle (Gibbs 2002; Geiselhardt et al. 2010). On the other hand, these features make them ideal for acting as recognition cues, since their fluidity and low melting points increase molecular mobility and volatility, facilitating detection by the sensory receptors of other insects and thus mediating intra- and interspecific recognition (Mazomenos et al. 2004; Beros et al. 2017). Chain length is another important factor in the biophysical properties of CHCs: the longer the chain, regardless of the presence of methyl groups or double bonds, the greater its waterproofing ability (Gibbs 1998; Sprenger et al. 2021). In this sense, the greater abundance of one type of CHC over another depends on several factors. Previous studies on flies have reported that CHCs can be influenced by various factors, such as genetic (Coyne et al. 1999; Foley et al. 2007; Sharma et al. 2012), environmental (Noorman and Otter 2002; Baleba et al. 2024; Kárpáti et al. 2023), dietary (Fedina et al. 2012), age (Vaníčková et al. 2012; Braga et al. 2016), and sex-specific factors (Gomes et al. 2008; Vaníčková et al. 2012; Butterworth et al. 2020).

As CHCs are generally species-specific, they can be used as chemotaxonomic markers, particularly for characterizing sibling species with subtle morphological differences (Howard et al. 1982; Golden et al. 1992; Etges and Jackson 2001). Regarding CHCs in Tephritoidea, there is limited literature available. Existing studies focus on tephritid fruit flies, such as *Anastrepha*, *Ceratitis*, and *Dacus* (Carlson and Yocom 1986; Sutton and Carlson 1993; Sutton and Steck 1994; Vaníčková et al. 2012). For Lonchaeidae, and particularly the genus *Neosilba*, to our knowledge, no studies involving CHCs have been conducted.

The genus *Neosilba* McAlpine (1962) (Lonchaeidae: Tephritoidea: Diptera) is restricted to the Neotropical region and is known to occur from the Caribbean, Mexico, and Colombia to Brazil. Forty species have been described, and at least 60 more await description (McAlpine and Steyskal 1982). *Neosilba* species are considered pests, using fruits of several economically significant dicotyledonous plants as hosts (Uchôa et al. 2002; Souza et al. 2005; Strikis and Prado 2005). The taxonomy of *Neosilba* species is a challenging task, as identification relies solely on the genita-l structures of male flies (McAlpine and Steyskal 1982). Furthermore, large cryptic species complexes within the genus *Neosilba* have been confirmed, and sometimes only unidentified species are reported (Uchôa et al. 2002; Bomfim et al. 2007).

Given the difficulties associated with the morphological identification of *Neosilba* species, we attempted to use CHCs to assist in their identification and differentiation. Moreover, as these species utilize many fruit species as host fruits, we also investigated whether the CHC patterns in *Neosilba* spp. vary across different host fruits.

## Methods and Materials

### Insects

We sampled host fruits from January 2011 to March 2013 in Paraibuna municipality, São Paulo state, southeastern Brazil (23°27’53.94”S, 45º42’31.88”W). Fruits were maintained until adult emergence following the procedures described by Uchôa and Zucchi (1999). Recently fallen or still attached ripe fruits showing signs of infestation (perforations or exudate) were collected in the field, botanically identified, and kept in individual trays covered with mesh (27 ± 1 °C; 70 ± 5% RH; 12 h L: D). Each batch received a unique code (fruit-lot ID) that was used throughout the entire process. Once larvae exited the fruit, they were transferred to sterilized rearing cups containing moist vermiculite. The emergence of each adult male was recorded under the same fruit-lot ID, ensuring that the host assigned to that individual corresponded to: (1) the oviposition choice of the female fly, as the egg was laid in that fruit; and (2) the larval diet, since all development occurred within that plant tissue.

We used six *Neosilba* species and only male flies for the cuticular hydrocarbon analyses, as traditional taxonomy in this group is based on the examination of male genitalia (McAlpine 1962; McAlpine and Steyskal 1982). Because genitalia dissection is destructive, all emerged adults were initially subjected to CHC extraction, after which specimens were sexed and identified to species level following McAlpine (1962) and McAlpine and Steyskal (1982). Following adult emergence, males were individually housed in labeled vials, with host fruit information maintained throughout the entire process. After CHC extraction, the same specimens were used for genitalia dissection and species confirmation, ensuring a one-to-one correspondence between chemical profiles, host fruit of origin, and taxonomic identification. Voucher specimens are deposited at the Universidade Estadual de Campinas (UNICAMP). The species sampled and their respective host fruits are listed in Table 1.

**Table 1:** Number of *Neosilba* males analysed per larval-host fruit

Neosilba species	Host fruit (common and scientific name)	Code	*n* males
*N. certa* (Walker)	Common guava *Psidium guajava* (Myrtaceae)	PGU	13
*N. glaberrima* (Wiedemann)	Common guava *Psidium guajava*	PGU	8
	Peach *Prunus persica* (Rosaceae)	PPE	5
*N. inesperata* (Strikis & Prado)	Peach *Prunus persica*	PPE	12
*N. pendula* (Bezzi)	Coffee *Coffea arabica* (Rubiaceae)	CAR	6
	Mulberry *Morus alba* (Moraceae)	MAL	5
	Peach *Prunus persica*	PPE	5
	Strawberry guava *Psidium cattleianum* (Myrtaceae)	PCA	5
	Cambuci *Campomanesia phaea* (Myrtaceae)	CPH	5
*N. perezi* (Romero & Ruppel)	Cassava *Manihot esculenta* (Euphorbiaceae)	MES	13
*N. zadolicha* (McAlpine)	Common guava *Psidium guajava*	PGU	5
	Juá *Zizyphus oblongis* (Rhamnaceae)	ZOB	4
	Negramina *Siparuna guianensis* (Siparunaceae)	SGU	8
	Araticum *Annona coriacea* (Annonaceae)	ACO	3

### Identification of Cuticular Hydrocarbons

We extracted CHCs dipping adult flies, individually, in 300 µL of *n*-hexane residue grade (J.T.Baker) for 10 min. We dried the extracts on N_2_ flux, recovered them with 10 µL *n*-hexane, and characterized the CHCs by injecting 1–5 µL sample by gas chromatography-mass spectrometry (GC-MS). We carried out the GC-MS analysis using a HP5890 gas chromatograph equipped with a fused silica capillary column containing a film of 5% diphenyl and 95% dimethylsiloxane (HP-5, 30 m x 0.25 mm x 0.25 μm), using He as the carrier gas (1.4 mL/min). We set the injector temperature at 270 °C in splitless mode. We set the initial oven temperature at 60 °C, increasing by 20 °C/min until 230 °C, and afterwards 3 °C min/1 to 300 °C, where the temperature was maintained for 5 min. We used a mass selective detector HP5973 in electron impact mode, at 70 ev, with the total ion current accumulated from m/z 40 to 600. We set the GC-MS interface temperature at 300 °C.

Cuticular hydrocarbons were identified based on retention index (RI) values calculated according to Van den Dool and Kratz (1963), using a homologous series of linear n-alkanes (C_7_-C_40_) as external standards, in combination with mass spectral fragmentation patterns. Compound identities were confirmed by comparison with reference spectra from the NIST mass spectral library (Carlson et al. 1998; Gomes et al. 2008). The position of double bonds in alkenes was determined after derivatization with dimethyl disulfide (DMDS), following established procedures and interpretation of diagnostic fragment ions (Carlson et al. 1989).

### Statistical Analysis

We calculated the relative abundance (%) of each cuticular hydrocarbon (CHC) by dividing the peak area of each compound by the total sum of all peak areas detected in the sample. CHCs with absolute abundances < 1% were excluded from the analysis.

We used Permutational Multivariate Analysis of Variance (PERMANOVA), with Bray-Curtis distance and 9999 permutations to: (1) assess whether the chemical cuticular profile of different *Neosilba* species is influenced by different host fruits and (2) whether there is intraspecific variation among individuals from different hosts. We also used Nonmetric Multidimensional Scaling (NMDS) to visualize graphically these differences. These statistical analyses used Past 3.20 software (Hammer 2001).

A random forest analysis was applied to the complete dataset, including all individuals, with the aim of identifying the cuticular hydrocarbon compounds most relevant for discriminating among fly species-host fruit combinations (*n* trees = 1000, *n* variables per split = 6). Compounds with high importance values (importance ≥ 0.9) identified in this analysis were subsequently subjected to separate Kruskal-Wallis (KW) tests to evaluate whether their relative abundances differed significantly among species associated with different host fruits (Firmino et al. 2020). All analyses were performed using Statistica 14 software (TIBCO 2020).

## Results

We identified 37 CHCs, ranging from C_23_ to C_33_, in 97 males of six species of *Neosilba* whose larvae feeding on 10 host fruits (see Table S1-S4). The monomethyl alkanes were the main CHCs with 20 compounds and only a single dimethyl alkane was present (5,11-DiMe-C_27_). Ten linear alkanes and six linear alkenes were also identified. Among the odd numbered backbone monomethyl alkanes we found the homologous series, ranging from C_23_ to C_29_, of 13-,11-,9-Me C_XX_, 7-Me-C_XX_, 5-Me-C_XX_, and 3-Me-C_XX_; the unusual 2-Me-C_27_ and 2-Me-C_29_ were also found. Moreover, we detected the even numbered monomethyl alkanes 12-, 10-Me-C_XX_, from C_24_ to C_28_, and 2-Me-C_30_. Among the alkanes, the homologous series 9-, and 7-Me-C_XX_, were detected from odd backbone C_23_ to C_29_.

**Fig. 1:**
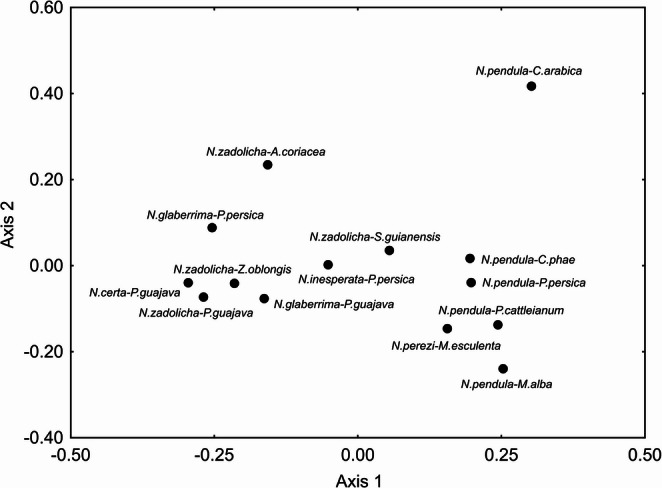
Non-metric multidimensional scaling (NMDS) plot based on the relative abundance of cuticular hydrocarbons from six *Neosilba* species reared from ten host fruits. Each point represents the mean of individual samples for each species-host fruit combination, and points closer together indicate more similar chemical profiles

Regarding the statistical analyses, PERMANOVA indicated significant differences in the cuticular profile between associations of *Neosilba* species with their respective host fruits (pseudo-F_(13, 3769)_ = 36.33, *p* = 0.0001). Complementing this analysis, NMDS ordination (Fig. 1) reveals that some groupings occurred according to both the species and the host fruit. For example, no significant intraspecific differences were observed in the cuticular profile of *N. glaberrima*,* N. pendula* and *N. zadolicha* in relation to their host fruits associated (*p* > 0.05). In *N. pendula*, the NMDS ordination suggests an apparent structuring according to host association, with a visual separation of individuals associated with *C. arabica*. However, this pattern was not supported by PERMANOVA, which detected no statistically significant differences among host-associated groups (*p* > 0.05). The fly *N. certa*, when associated with *P. guajava*, showed significant differences in the cuticular profile in relation to the most of other species (*p* < 0.05), except in comparison with *N. glaberrima* associated with *P. persica* and *N. zadolicha* associated with *A. coriacea*, *P. guajava* and *Z. oblongis* (*p* > 0.05). Similarly, *N. inesperata* associated with *P. persica* showed significant differences in relation to all species (*p* < 0.05), except for *N. zadolicha* associated with *P. guajava*, *S. guianensis* and *A. coriacea*. Likewise, *N. perezi* associated with *M. esculenta* showed significant differences in the cuticular profile in relation to all other species, except *N. pendula* associated to *P. cattleianum* and *P. persica* and *N. zadolicha* associated with *S. guianensis* and *A. coriacea*, with which no significant differences were observed (*p* > 0.05). Regarding the host fruit *P. guajava*, the NMDS ordination shows an overlap among *N. certa*, *N. glaberrima*, and *N. zadolicha*, indicating overall similarity in their cuticular hydrocarbon profiles when associated with this host (Fig. 1). Nevertheless, pairwise PERMANOVA revealed a significant difference between *N. certa*-*P. guajava* and *N. glaberrima*-*P. guajava* (*p* < 0.05), whereas neither species differed significantly from *N. zadolicha*-*P. guajava* (*p* > 0.05). Regarding the host fruit *P. persica*, the NMDS ordination did not show visual overlap among *N. glaberrima*, *N. pendula*, and *N. inesperata*, indicating a lack of apparent similarity in their cuticular hydrocarbon profiles when associated with this host (Fig. 1). Nevertheless, pairwise PERMANOVA revealed no significant difference between *N. glaberrima*-*P. persica* and *N. pendula*-*P. persica* (*p* > 0.05), whereas both differed significantly from *N. inesperata*-*P. persica* (*p* < 0.05). All p-values ​​from the a posteriori PERMANOVA test are available in Supplementary Table S5.

**Fig. 2:**
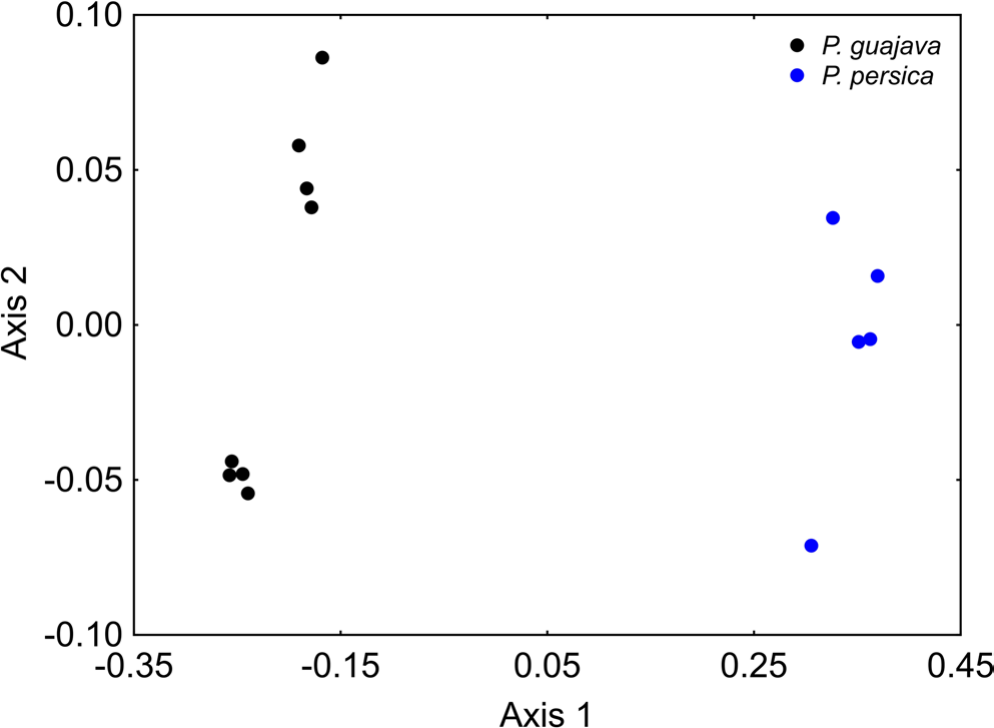
NMDS plot showing the differences in cuticular hydrocarbon composition of *N. glaberrima* reared from *P. guajava* and *P. persica* fruits. Each point represents an individual sample, and proximity indicates similarity in chemical profiles

**Fig. 3:**
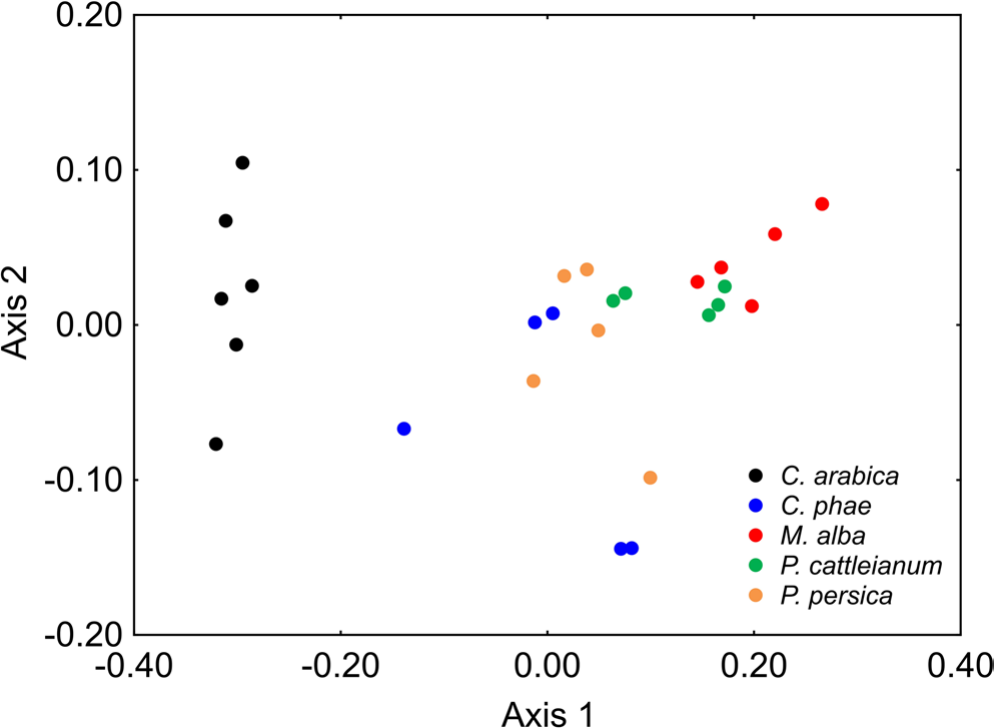
NMDS plot showing the variation in cuticular hydrocarbon composition of *N. pendula* reared from five host fruits: *C. arabica*, *C. phae*, *M. alba*, *P. cattleianum*, and *P. persica*. Each point represents an individual sample, and proximity among points indicates similarity in chemical profiles. Formal statistical comparisons among host-associated groups are provided in Table S6

**Fig. 4:**
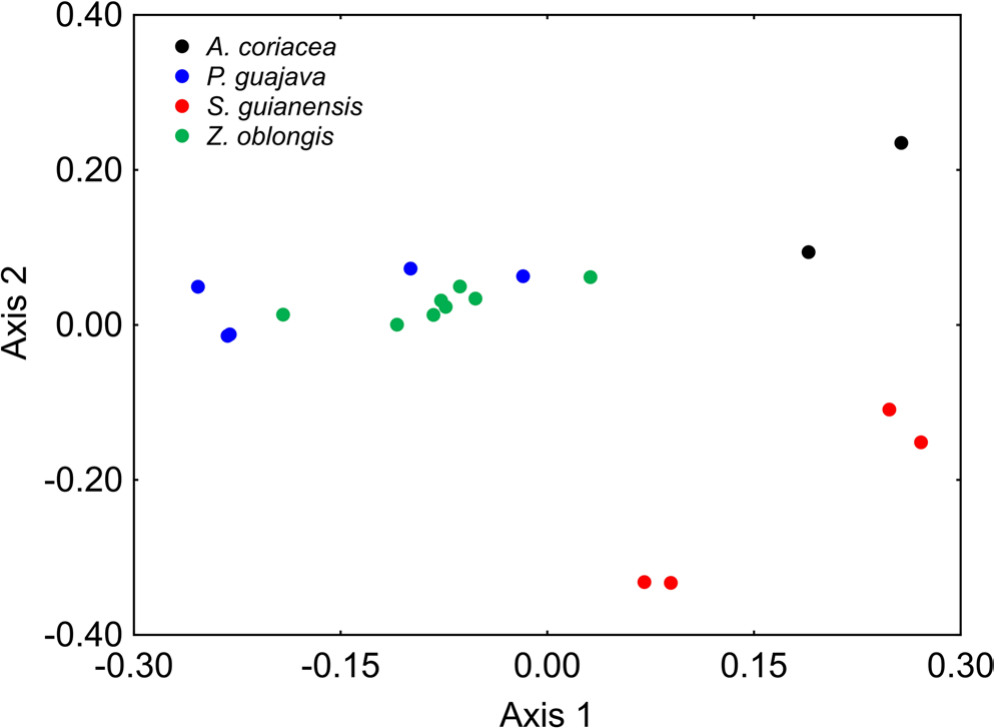
NMDS plot showing the variation in cuticular hydrocarbon composition of *N. zadolicha* reared from four host fruits: *A. coriacea*, *P. guajava*, *S. guianensis*, and *Z. oblongis*. Each point represents an individual sample, and proximity among points indicates similarity in chemical profiles. The overlap observed among some host fruit groups reflects similarity in chemical composition, and formal statistical comparisons are provided in Table S7

When PERMANOVA was applied separately to fly species with multiple hosts, significant differences were found for *N. glaberrima* (pseudo-F_(1, 206)_ = 37.47, *p* = 0.0006), *N. pendula* (pseudo-F_(4, 489)_ = 30.90, *p* = 0.0001), and *N. zadolicha* (pseudo-F_(3, 336)_ = 15.26, *p* = 0.0001) among their respective host fruits (Figs. 2, 3 and 4). However, some patterns deserve closer attention. For instance, a result like that observed in Fig. 1 was detected for *N. pendula* in Fig. 3, where individuals associated with different host fruits showed similar cuticular profiles (*p* > 0.05), except those reared from *C. arabica*, which differed significantly from the others (*p* < 0.05) (Fig. 3; see PERMANOVA results in Table S6). For *N. zadolicha*, pairwise PERMANOVA revealed host-associated differences in cuticular hydrocarbon profiles among specific host fruit comparison. Significant differences were detected between individuals associated with *P. guajava* and *S. guianensis*, between *P. guajava* and *Z. oblongis*, and between *S. guianensis* and *Z. oblongis*, as well as between *Z. oblongis* and *A. coriacea* (*p* < 0.05). In contrast, no significant differences were observed between *P. guajava* and *A. coriacea*, nor between *S. guianensis* and *A. coriacea* (*p* > 0.05) (Fig. 4; see PERMANOVA results in Table S7).

Random forest analysis identified four hydrocarbons with importance ≥ 0.90 for discriminating *Neosilba* samples from their respective hosts. The compound 13-,11-,9-Me C_23_ (Retention Index, RI = 2337; importance = 1.00) had the highest weight in the model, separating *N. certa-P. persica* from *N. pendula* (from all hosts) and from *N. perezi-M. esculenta* (*p* < 0.05, KW test). It also distinguished *N. glaberrima-P. persica* from *N. pendula* associated with *C. phae*, *M. alba*, *P. cattleianum*, and *N. perezi* (*p* < 0.05, KW test). The hydrocarbon 8-Me C_24_ (RI = 2438; importance = 0.99) was relevant in separating *N. inesperata-P. persica* in relation to *N. certa-P. guajava*, *N. perezi-M. esculenta*, and *N. pendula* associated with *C. phae*, *M. alba*, and *P. cattleianum* (*p* < 0.05, KW test).

The compound C_25_ (RI = 2500; importance = 0.95) showed significant differences within *N. pendula*, distinguishing individuals associated with *C. arabica* from the other populations analyzed (*p* < 0.05, KW test), except from those associated with *C. phaea* (*p* > 0.05, KW test). Finally, the hydrocarbon 12-,10-Me C_26_ (RI = 2634; importance = 0.92) distinguished *N. certa-P. guajava* from *N. perezi-M. esculenta* and from *N. pendula* associated with *P. cattleianum*, *M. alba*, and *P. persica* (*p* < 0.05, KW test). We emphasize that only the main separations are presented in this text; the other results and complementary values ​​of the random forest analysis are available in the Supplementary Material (file Random_Forest_Neosilba.xlsx).

## Discussion

Species identification is fundamental to understanding the biology and ecology of insect populations and their impacts on resources. In particular, phytophagous flies are often composed of sibling species or species complexes with uncertain identities and subtle ecological differences (Rossi et al. 1999; Tabuchi and Amano 2003). Failure to separate these closely related species and recognize their different biological adaptations can create serious problems in pest management (Doellman et al. 2020; Wang et al. 2024).

Both classical taxonomic methods and biosystematics studies have been powerful tools for revealing cryptic species. However, the phenotype of an organism also includes sets of chemical traits, such as CHCs, which provide additional information to unravel complex taxonomic relationships (Carlson 1988; Golden et al. 1992; Gemeno et al. 2012).

Nonetheless, our study on CHCs in six *Neosilba* species indicates that cuticular hydrocarbons alone do not provide a fully reliable taxonomic tool for distinguishing species within the genus. However, our findings also suggest that CHC profiles can serve as valuable complementary evidence when specific compounds are considered. Among Tephritidae, the use of CHCs as taxonomic tools has shown mixed results. For example, Lavine et al. (1992) successfully differentiated the larvae of *Anastrepha fraterculus*, *A. suspensa*, and *A. acris*, but *A. suspensa* could not be differentiated from *A. acris*. However, Goh et al. (1993) were able to separate species of the genus *Bactrocera*, which are closely related and morphologically indistinguishable. A similar result was reported by Vaníčková et al. (2014), who successfully used CHCs as a taxonomic tool for three cryptic species of flies in the genus *Ceratitis*.

Although CHCs can be a complementary tool for species identification, they also present certain limitations (Kather and Martin 2012; Buellesbach et al. 2018). According to Kather and Martin (2012), factors such as temperature and diet can alter the cuticle, influencing the presence or absence of certain compounds, which could lead to incorrect species identification. Differences in the abundance or number of CHCs in the cuticle do not necessarily imply that they belong to different species. In this sense, it is worth noting that the experimental design of previously published studies (Lavine et al. 1992; Goh et al. 1993; Vaníčková et al. 2014) differs from ours, as those studies used flies from laboratory colonies under controlled conditions.

We observed significant differences in the cuticular profile among associations of *Neosilba* species with their respective host fruits. However, some groupings occurred according to the species or the host fruit. We found consistent clustering of *N. glaberrima* and *N. zadolicha* based on species, regardless of the host fruit. *N. certa*, *N. inesperata*, and *N. perezi*, which were associated with a single host, showed cuticular profiles similar to those of different species associated with different hosts. According to the literature, genetic and ecological causes are the main explanations for this result (Jallon and David 1987; Coyne 1996). Jallon and David (1987) observed a similar pattern when they detected consistent similarities in the cuticular profiles of different *Drosophila* species. According to the authors, this similarity is probably due to their close phylogenetic relationships and shared ecological adaptations, explained by the fact that they are geographically and phylogenetically related or share similar habitats and ecological niches, which may select for comparable chemical signals. The authors emphasize that these species tend to be almost identical in their chemical profiles, reflecting their close genetic ties and possible conserved mechanisms of chemical communication.

Recent studies on lonchaeid flies associated with figs have shown that *Neosilba* populations often form species complexes, in which molecular data reveal several well-supported clades that are only partially distinguishable based on morphology (Lasa et al. 2025a; MacGowan et al. 2025). This reinforces the idea that the current taxonomy of the genus is still provisional and that cryptic diversity is likely extensive. Our CHC profiles are consistent with this scenario: even within morphologically identified species, we observed structured variation among populations, suggesting that at least part of the “intraspecific” chemical variation may reflect mixtures of closely related lineages that have not yet been formally described. Therefore, we interpret our results with caution, recognizing both the ecological signal captured by the CHCs and the taxonomic instability that still characterizes *Neosilba*.

We also observed a visual inspection of the NMDS ordination revealed apparent host-associated clustering for some species, including *N. certa*, *N. glaberrima*, and *N. zadolicha* associated with *P. guajava* (Fig. 1). However, PERMANOVA provided only partial statistical support for this pattern, as a significant difference was detected between *N. certa* and *N. glaberrima*, whereas neither species differed significantly from *N. zadolicha* (*p* > 0.05, see Table S5). Similarly, we observed significant diet-related differences when PERMANOVA was applied separately to flies with multiple hosts, such as *N. pendula*, which showed associations with nearly all host species, except *C. arabica* (Fig. 3). The host fruits influenced the cuticular profile to the extent that flies of the same species on different host fruits exhibited distinct cuticular differences, expressing certain unique compounds (Tables S2-S4). In the literature, diet is reported as one of the main environmental factors that can influence the cuticular hydrocarbon profiles of insects and, consequently, intraspecific recognition (Geiselhardt et al. 2012; Valadares et al. 2015; Merli et al. 2018; Blomquist and Ginzel 2021). The study by Geiselhardt et al. (2012) supports this relationship between diet and CHC profile, demonstrating that the cuticular hydrocarbons of the beetle species *Phaedon cochleariae* vary significantly depending on the host fruit used. According to the authors, males prefer females fed on the same host fruit, a behavior mediated by CHCs. This phenotypic plasticity in mating preferences may act as an initial barrier to gene flow between populations associated with different hosts, promoting behavioral isolation and ecological speciation.

Additionally, Otte et al. (2016) demonstrated that such phenotypic plasticity could also reduce sexual interference between sympatric species. In the case of the beetles *Phaedon cochleariae* and *P. armoraciae*, when fed on the same host, differences in CHCs were minimized, resulting in interspecific mating and the loss of behavioral isolation. This result indicates that shared hosts may lead to convergence in partner recognition systems, enabling these species to coexist in sympatry without significant genetic divergence. However, when each species was maintained on its natural host, CHCs diverged, and reproductive isolation was restored.

In flies, the correlation between CHCs and host fruits was demonstrated by Stennett and Etges (1997), who showed that *Drosophila mojavensis* and *D. arizonae* exhibited CHCs dependent on the larval diet. In the Tephritidae family, Hood et al. (2022) demonstrated that when different species of flies from the genus *Rhagoletis* used the same host, overlap in chemical profiles could lead to confusion in partner recognition. This could increase the chances of interspecific mating, as the chemical cues that typically facilitate species-specific mate choice may become less distinct. Complementarily, Geiselhardt et al. (2012) proposed that the host fruit can impact the biosynthesis of CHCs, directly influencing sexual recognition signals and leading to the formation of reproductive isolations without prior genetic divergence. The host-dependent CHC expression found in our study may suggest the formation of host races, a population of a species living and showing preference for a host different from the host(s) of other populations of the same species (Bush 1969; Drès and Mallet 2002; Cha et al. 2012; Ragland et al. 2012).

An interesting result was observed for *N. pendula* associated with *C. arabica*, which showed a distinct cuticular profile compared to other hosts of the same species (Fig. 3). The cuticle of *N. pendula* associated with *C. arabica* exhibited a higher abundance of certain compounds relative to other hosts of the same species; for instance, C_25_ and 13-,11-,9-Me C_25_ had relative abundances of 30.84% and 44.59% on the cuticle, respectively, whereas the maximum percentages observed for other hosts of *N. pendula* were 11.19% and 31.35%, respectively (Table S3). In this context, Fernandes et al. (2012) observed that nutrients and secondary metabolites present in *C. arabica* can influence the performance and behavior of the hemipteran *Coccus viridis*. Behavioral changes due to contact with caffeine were also observed in *Apis mellifera* (Couvillon et al. 2015). According to Mustard (2014), this occurs because caffeine affects insect metabolism and physiology, altering the expression of genes involved in lipid metabolism and, consequently, the composition of cuticular hydrocarbons, since these compounds depend on lipid and enzymatic pathways, resulting in a differentiated chemical profile. In summary, the chemical profile of the host fruit shapes the insect’s response, whether at the physiological or behavioral level. This is particularly relevant because, in *Neosilba*, a genus with complex identification, such host-induced changes can blur species delimitations based on CHCs.

Random forest analysis indicated that only a few compounds had high importance in discriminating *Neosilba* individuals and their respective hosts. The compound 13-,11-,9-Me C_23_ had a relative abundance of about 40% in *N. certa-P. persica*, while it was absent in *N. perezi-M. esculenta* and ranged from 0.93% to 3.24% in *N. pendula*. The hydrocarbon 8-Me C_24_ was detected at 2.99% in *N. inesperata* but was absent in the other species. The compound C_25_ had a high relative abundance of 30.84% in *N. pendula-C. arabica*, contrasting with the lower values observed in other populations of the same species (3.25% to 6.45%), except for *N. pendula-C. phae*, which reached 11.19%.

However, these differences appear to primarily reflect the influence of the host fruit rather than genetic divergence among species. Therefore, future studies on cuticular hydrocarbons in *Neosilba* should consider the host fruit as a central factor of variation, as it may obscure or mimic genuine interspecific differences. This aspect is particularly relevant in a genus that continues to expand its range of hosts, with new records of associations with different fruits (Lemos et al. 2015; Gisloti et al. 2017; Vieira et al. 2019; Sousa et al. 2021; Barreto et al. 2023; Canejo et al. 2023; Coelho and Uchoa 2023; Lasa et al. 2025a, 2025b).

Although host fruit can influence CHC profiles, we cannot discard the influence of abiotic factors, such as temperature and humidity, as this study was conducted in the field, where flies were exposed to similar environmental conditions. In the literature, it has been demonstrated that insects exposed to comparable abiotic conditions tend to exhibit similar chemical profiles as well (Michelutti et al. 2018; Duarte et al. 2019; Santos-Junior et al. 2022; Baleba et al. 2024; Lima et al. 2025).

This study represents the first attempt to unravel the cuticular chemistry of the genus *Neosilba*, exploring how CHC profiles vary among species and their host fruits. Although the results indicate that these compounds alone cannot yet be considered definitive taxonomic markers, they reveal intriguing patterns of variation that may reflect both phylogenetic relatedness and ecological adaptations. In particular, although ecological factors such as host fruit can influence certain characteristics of the cuticular hydrocarbon profile of *Neosilba*, these effects do not obscure species-specific chemical differences. These findings reveal unprecedented patterns in the chemical ecology of *Neosilba*, suggesting that CHCs may function as complementary, though not conclusive, characters in species delimitation.

Given the remarkable plasticity of cuticular hydrocarbons and their sensitivity to environmental factors, future studies integrating molecular, ecological, and chemical approaches will be essential to uncover the true taxonomic and evolutionary signals, including the speciation processes, underlying these patterns. In this sense, the analysis of cuticular hydrocarbons in *Neosilba* may serve as a complementary character for species delimitation, although its limitations indicate that it is not sufficient by itself for definitive identification. This reflects the subtle connections between adaptation, speciation, and taxonomy in these enigmatic dipterans.

## Supplementary Information

Below is the link to the electronic supplementary material.

Supplementary File 1 (XLSX 34.4 KB) 

Supplementary File 2 (XLSX 107 KB)

Supplementary File 3 (PDF 369 KB)

## Data Availability

No datasets were generated or analysed during the current study.
