# Supplementary material for: Can Cuticular Hydrocarbons Be used as Chemotaxonomic Tool for Neosilba McAlpine (Diptera: Lonchaeidae)?
Source: J Chem Ecol. 2026 Feb 24;52(2):22. doi: 10.1007/s10886-026-01693-8 (PMC12932378; doi:10.1007/s10886-026-01693-8)
Supplement: Supplementary file 3 — (PDF 369 KB) [file 10886_2026_1693_MOESM3_ESM.pdf]

Table S1. Cuticular hydrocarbon composition of *Neosilba certa* using *Psidium guajava* as host plant, *Neosilba inesperata* using *Prunus persica*, and *Neosilba perezi* using *Manihot esculenta*.

RI	Compound	Relative abundance (%) (mean ± SD)		
		N. certa (n = 13)	*N. inesperata* (n = 14)	*N. perezi* (n = 15)
2300	C_{23}	7.44±2.25	2.95±0.98	-
2336	13-,11-,9-Me C_{23}	40.33±9.60	21.38±5.87	-
2348	5-Me C_{23}	-	1.78±0.83	-
2571	3-Me C_{23}	-	0.82±1.06	-
2400	C_{24}	1.19±0.59	0.79±0.76	-
2432	10-Me C_{24}	1.95±0.37	-	-
2438	8-Me C_{24}	-	2.99±0.89	-
2443	6-Me C_{24}	-	0.46±0.64	-
2481	$C_{25:1}$	-	1.20±1.16	-
2500	C_{25}	9.59±3.77	8.67±2.80	7.38±1.26
2538	13-,11-,9-Me C_{25}	3.90±0.70	14.94±5.69	16.30±7.48
2541	7-Me C_{25}	-	4.32±4.00	-
2549	5-Me C_{25}	-	1.68±0.42	-
2573	3-Me C_{25}	0.57±0.50	1.45±1.67	0.95±0.77
2600	C_{26}	0.64±0.49	1.42±0.34	1.01±0.51
2631	12-,10-Me C_{26}	0.10±0.36	0.99±0.70	2.87±1.07
2655	4-Me C_{26}	-	0.09±0.34	-
2671	$C_{27:1}$	-	-	0.52±0.80
2682	$C_{27:1}$	0.57±0.49	0.28±0.55	1.70±1.02
2700	C_{27}	13.70±5.17	11.66±1.60	11.80±5.69
2731	13-,11-,9-Me C_{27}	3.84±3.10	6.50±2.61	25.48±4.53
2737	7-Me C_{27}	0.82±0.77	1.19±0.87	0.82±0.83
2760	2-Me C_{27}	0.25±0.48	0.10±0.38	1.15±1.12
2771	3-Me C_{27}	2.53±0.68	2.93±0.74	2.86±1.16
2800	C_{28}	1.08±0.55	0.97±0.59	0.45±0.65

RI	Compound	Relative abundance (%) (mean ± SD)		
		N. certa (n = 13)	*N. inesperata* (n = 14)	*N. perezi* (n = 15)
2836	12-,10-Me C_{28}	-	-	2.46±1.19
2860	2-Me C_{28}	0.35±0.73	-	-
2873	$C_{29:1}$	0.50±0.62	0.09±0.33	2.69±0.98
2883	$C_{29:1}$	1.58±0.92	0.69±0.64	6.08±1.43
2900	C_{29}	3.45±1.49	2.97±0.93	3.56±1.99
2928	13-,11-,9-Me C_{29}	1.55±1.10	2.95±1.34	4.80±1.44
2934	7-Me C_{29}	0.06±0.20	0.31±0.89	0.89±1.71
2971	3-Me C_{29}	3.63±1.28	3.46±1.60	6.22±1.75

Table S2. Cuticular hydrocarbon composition of *Neosilba glaberrima* using as host plants *Psidium guajava* or *Prunus persica*.

RI	Compound	Relative abundance (%) (mean ± SD)	
		Psidium guajava (n = 10)	*Prunus persica* (n = 5)
2300	C_{23}	4.59±0.38	6.79±1.23
2336	13-,11-,9-Me C_{23}	38.14±7.08	29.67±1.61
2571	3-Me C_{23}	-	0.29±0.64
2400	C_{24}	0.58±0.68	2.04±0.11
2432	10-Me C_{24}	2.53±0.08	3.33±0.28
2500	C_{25}	6.69±0.68	16.51±0.51
2538	13-,11-,9-Me C_{25}	10.88±0.55	8.61±0.65
2541	7-Me C_{25}	0.47±0.76	1.23±0.71
2573	3-Me C_{25}	0.39±0.41	2.10±0.10
2600	C_{26}	-	1.54±0.13
2631	12-,10-Me C_{26}	0.07±0.22	-
2682	7:$C_{27:1}$	1.15±0.46	-
2700	C_{27}	9.86±2.34	16.80±0.55
2731	13-,11-,9-Me C_{27}	6.18±1.87	1.02±0.93
2737	7-Me C_{27}	1.26±0.54	-
2760	2-Me C_{27}	0.43±0.55	-
2771	3-Me C_{27}	2.39±0.89	0.21±0.48
2800	C_{28}	0.72±0.34	1.03±0.58
2900	C_{29}	3.42±0.75	4.65±0.21
2928	13-,11-,9-Me C_{29}	2.79±0.38	-
2934	7-Me C_{29}	0.08±0.24	-
2971	3-Me C_{29}	2.74±0.32	1.59±0.07
3052	2-Me C_{30}	-	2.59±0.16

Table S3. Cuticular hydrocarbon composition of *Neosilba pendula* using as host plants *Coffea arabica*, *Psidium cattleianum*, *Campomanesia phaea*, *Morus alba* or *Prunus persica*.

RI	Compound	Relative abundance (%) (mean ± SD)				
		Coffea arabica (n = 6)	*Psidium cattleianum* (n = 5)	*Campomanesia phaea* (n = 5)	*Morus alba* (n = 5)	*Prunus persica* (n = 5)
2300	C_{23}	0.19±0.46	0.52±0.75	2.25±0.96	0.87±1.41	2.66±1.72
2336	13-,11-,9-Me C_{23}	2.72±0.79	0.95±0.63	2.35±1.26	0.93±1.24	3.24±1.12
2371	Unknown	-	0.35±0.50	0.44±0.98	0.53±1.17	0.27±0.61
2571	3-Me C_{23}	-	0.20±0.46	1.72±2.38	-	1.26±1.35
2400	C_{24}	1.22±0.85	0.30±0.41	0.94±0.94	-	0.55±0.76
2432	10-Me C_{24}	1.78±0.34	0.83±0.52	2.54±1.06	0.47±0.44	2.10±0.60
2481	$C_{25:1}$	-	-	0.92±0.97	0.58±0.96	0.58±1.29
2500	C_{25}	30.84±8.16	4.85±1.50	11.19±4.61	3.25±0.55	6.45±1.82
2538	13-,11-,9-Me C_{25}	44.59±10.55	19.72±6.17	26.12±10.38	11.92±6.01	31.35±2.13
2573	3-Me C_{25}	7.79±0.89	1.07±1.12	2.45±0.90	0.35±0.49	1.90±0.41
2600	C_{26}	1.66±0.61	1.48±0.42	1.63±1.12	0.88±0.58	1.33±0.78
2631	12-,10-Me C_{26}	0.61±0.52	2.06±1.15	2.28±0.15	2.80±0.30	2.85±0.24
2671	9:$C_{27:1}$	-	0.49±0.44	-	-	-
2682	7:$C_{27:1}$	-	0.99±0.72	0.75±0.45	0.65±0.37	0.17±0.37
2700	C_{27}	0.30±0.48	10.07±1.79	13.13±4.65	9.99±0.96	9.57±0.72
2731	13-,11-,9-Me C_{27}	0.78±0.45	29.90±3.63	16.79±3.62	39.40±3.76	19.43±3.50
2737	7-Me C_{27}	-	0.17±0.39	-	-	-
2760	2-Me C_{27}	-	0.30±0.41	-	0.28±0.39	-
2771	3-Me C_{27}	-	3.19±0.21	3.32±0.97	3.32±0.56	2.29±1.40
2781	5,11-DiMe C_{27}	-	-	-	0.47±0.73	0.46±0.74
2800	C_{28}	-	0.44±0.60	0.64±0.87	0.39±0.35	0.46±0.63
2836	12-,10-Me C_{28}	-	2.63±0.77	0.52±0.72	2.96±0.61	1.15±1.05
2860	2-Me C_{28}	-	0.13±0.29	-	0.16±0.35	-
2873	9-$C_{29:1}$	-	1.79±0.46	0.45±0.66	1.42±1.02	0.37±0.55
2883	7-$C_{29:1}$	-	1.98±1.12	0.89±0.82	2.98±0.40	1.39±0.78

RI	Compound	Relative abundance (%) (mean ± SD)				
		Coffea arabica (n = 6)	*Psidium cattleianum* (n = 5)	*Campomanesia phaea* (n = 5)	*Morus alba* (n = 5)	*Prunus persica* (n = 5)
2900	C_{29}	-	1.83±0.35	1.89±1.49	1.50±0.95	2.00±0.37
2928	13-,11-,9-Me C_{29}	-	8.49±2.68	3.40±1.03	9.22±1.45	5.35±2.58
2934	7-Me C_{29}	-	0.33±0.45	-	-	-
2971	3-Me C_{29}	-	4.69±0.58	3.40±1.76	4.66±0.76	2.82±0.54
3100	C_{31}	1.11±0.99	0.22±0.50	-	-	-
3173	3-Me C_{31}	0.13±0.31	-	-	-	-
3200	C_{32}	0.86±0.69	-	-	-	-
3300	C_{33}	5.43±2.12	-	-	-	-

Table S4. Cuticular hydrocarbon composition of *Neosilba zadolicha* using as host plants *Psidium guajava, Siparuna guianensis, Zizyphus oblongis* or *Annona coriacea.*

RI	Compound	Relative abundance (%) (mean ± SD)			
		Psidium guajava (n=5)	*Siparuna guianensis* (n=4)	*Zizyphus oblongis* (n=8)	*Annona coriacea* (n = 3)
2300	C_{23}	4.44±0.50	1.72±0.82	8.31±2.76	4.54±1.70
2336	13-, 11, 9-Me C_{23}	42.90±8.31	9.89±4.44	33.33±11.49	12.60±4.86
2571	3-Me C_{23}	0.93±0.61	-	0.72±1.74	-
2400	C_{24}	0.57±0.32	0.45±0.52	1.09±1.19	1.46±0.81
2432	10-Me C_{24}	2.58±0.67	1.85±1.65	2.99±1.11	1.27±0.35
2481	Unknown	-	0.38±0.44	0.10±0.28	-
2500	C_{25}	7.96±0.58	8.17±2.28	7.91±1.50	11.97±7.25
2538	13-, 11-, 9-Me C_{25}	9.91±2.27	33.32±9.15	8.18±1.50	4.26±1.62
2573	3-Me C_{25}	0.38±0.57	0.66±0.76	-	-
2600	C_{26}	0.64±0.71	0.76±0.10	-	1.47±0.44
2631	12-,10-Me C_{26}	-	1.56±0.18	-	-
2671	9:$C_{27:1}$	-	0.46±0.53	-	-
2682	7:$C_{27:1}$	0.13±0.29	1.14±0.38	0.34±0.75	-
2700	C_{27}	10.96±3.85	11.97±6.10	14.16±2.96	28.70±3.32
2731	13-, 11-, 9-Me C_{27}	5.11±0.57	8.69±1.27	5.81±1.55	4.12±2.99
2760	2-Me C_{27}	0.68±0.93	0.34±0.39	0.09±0.26	-
2771	3-Me C_{27}	2.06±0.16	2.82±1.17	1.82±1.20	2.55±0.43
2800	C_{28}	0.94±0.32	0.76±0.91	0.69±0.74	2.89±0.68
2836	12-,10-Me C_{28}	-	0.24±0.48	-	-
2860	2-Me C_{28}	1.77±2.42	0.19±0.38	-	0.60±0.54
2873	9-$C_{29:1}$	-	1.54±1.09	0.86±0.87	0.41±0.72
2883	7-$C_{29:1}$	0.56±0.54	1.67±0.40	2.12±0.63	0.67±0.65
2900	C_{29}	2.97±1.88	5.16±3.44	4.72±1.27	12.93±2.22

RI	Compound	Relative abundance (%) (mean ± SD)			
		Psidium guajava (n=5)	*Siparuna guianensis* (n=4)	*Zizyphus oblongis* (n=8)	*Annona coriacea* (n = 3)
2928	13-,11-, 9-Me C_{29}	2.12±1.48	2.46±0.82	2.73±0.77	1.52±1.50
2960	2-Me C_{29}	-	-	-	0.30±0.52
2971	3-Me C_{29}	1.67±0.98	3.80±2.16	4.02±1.49	7.74±0.67

Table S5. Pairwise PERMANOVA *p*-values based on Bray-Curtis dissimilarities of cuticular hydrocarbon profiles among six *Neosilba* species reared from ten host fruits. Values indicate the probability of no significant difference between the chemical profiles of each species-host combination. Red-highlighted cells indicate statistically significant pairwise differences ($p < 0.05$).

	N.certa-P.guajava	*N.glaberrima-P. guajava*	*N.glaberrima-P. persica*	*N.inesperata-P. persica*	*N.pendula-C.arabica*	*N.pendula-P.cattleianum*	*N.pendula-C. phaea*	*N. pendula-M. alba*	*N.pendula-P.persica*	*N. perezi-M.esculenta*	*N.zadolicha-P. guajava*	*N.zadolicha-S.guianensis*	*N. zadolicha-Z. oblongis*
N. glaberrima-P. guajava	0.0091												
N. glaberrima-P. persica	0.0910	0.0910											
N. inesperata-P. persica	0.0091	0.0091	0.0273										
N. pendula-C. arabica	0.0182	0.0364	0.1456	0.0091									
N. pendula-P. cattleianum	0.0091	0.0637	0.8008	0.0273	0.2366								
N. pendula-C. phaea	0.0091	0.0728	0.8008	0.0364	0.1911	0.7007							
N. pendula-M. alba	0.0182	0.0637	0.7189	0.0273	0.2821	1.0000	0.7553						
N. pendula-P. persica	0.0182	0.0728	0.7280	0.0182	0.1729	1.0000	1.0000	0.6734					
N. perezi-M. esculenta	0.0091	0.0091	0.0364	0.0091	0.0182	1.0000	0.0182	0.0091	0.0637				
N. zadolicha-P. guajava	1.0000	1.0000	0.6916	0.0546	0.1729	0.6825	0.8190	0.7007	0.6552	0.0182			
N. zadolicha-S. guianensis	0.0455	0.1820	0.6916	0.4823	0.4641	0.7007	1.0000	0.6734	0.8554	0.0637	0.7098		
N. zadolicha-Z. oblongis	0.7553	0.1183	0.0455	0.0091	0.0637	0.0728	0.0728	0.0637	0.0728	0.0091	1.0000	0.1820	
N. zadolicha-A. coriacea	0.1820	0.6552	1.0000	0.1911	0.9100	1.0000	1.0000	1.0000	1.0000	0.1274	1.0000	1.0000	0.5278

Table S6. Pairwise PERMANOVA p-values based on Bray-Curtis dissimilarities of cuticular hydrocarbon profiles for *Neosilba pendula* reared from different host fruits. Red-highlighted cells correspond to statistically significant pairwise comparisons ($p < 0.05$).

	N. pendula-C. arabica	*N. pendula-P. cattleianum*	*N. pendula-C. phae*	*N. pendula-M. alba*
N. pendula-P. cattleianum	0.0260			
N. pendula-C. phae	0.0160	0.0710		
N. pendula-M. alba	0.0250	0.3980	0.0750	
N. pendula-P. persica	0.0200	0.1810	0.8200	0.0860

Table S7. Pairwise PERMANOVA p-values based on Bray-Curtis dissimilarities of cuticular hydrocarbon profiles for *Neosilba zadolicha* reared from different host fruits. Red-highlighted cells correspond to statistically significant pairwise comparisons ($p < 0.05$).

	N. zadolicha-P. guajava	*N. zadolicha-S. guianensis*	*N. zadolicha-Z. oblongis*
N. zadolicha-S. guianensis	0.0372		
N. zadolicha-Z. oblongis	0.2916	0.0114	
N. zadolicha-A. coriacea	0.1200	0.1644	0.0354

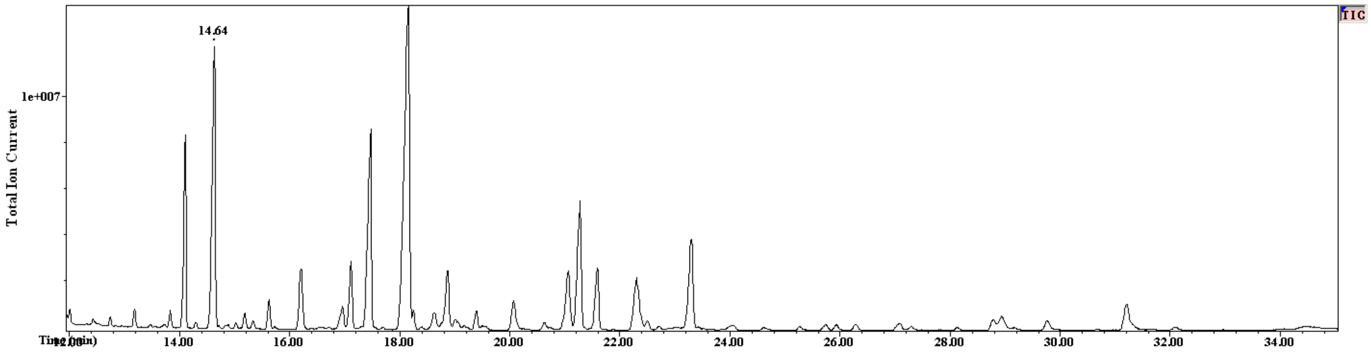

Figure S1. Representative cuticular hydrocarbon chromatogram of *Neosilba perezi*. This is the only chromatogram currently available, as the original diagnostic ion files could not be recovered.
